# Advancing Inclusive Aging and Dementia Research: Community Engagement and Recruitment Strategies at the Healthy Aging and Alzheimer's Research Care (HAARC) Center

**DOI:** 10.1002/alz70860_107156

**Published:** 2025-12-23

**Authors:** Phyllis Timpo, Rhiana Schafer, Hanna Clem, Chandler Zolliecoffer, Sophia Moore, Emily Kaderabek, Kaitlin Seibert, Kianna Hearns, Miles Breese, Hannah Whitmore, Rebecca Devine, Emily Cummings, Adam Martersteck, Emily J Rogalski

**Affiliations:** ^1^ University of Chicago, Chicago, IL, USA; ^2^ Healthy Aging & Alzheimer's Research Care (HAARC) Center, University of Chicago, Chicago, IL, USA; ^3^ Healthy Aging and Alzheimer's Research Center, University of Chicago, Chicago, IL, USA; ^4^ Healthy Aging and Alzheimer's Research Care Center, University of Chicago, Chicago, IL, USA; ^5^ Healthy Aging and Alzheimer's Research Care Center, Chicago, IL, USA

## Abstract

**Background:**

The University of Chicago's Healthy Aging & Alzheimer's Research Care (HAARC) Center, established in 2023, serves as a hub for advancing research on aging, Alzheimer's disease, and related dementias (ADRD), with a focus on resilience, resistance, and extending health span. Recognizing that meaningful progress in ADRD research requires representative perspectives, community engaged research (CER) has been integral to our approach. This report outlines CER and recruitment strategies over 18 months.

**Method:**

Multiple CER efforts were deployed including; hiring diverse study staff, establishing bi‐directional community partnerships, integrating participant and community‐informed feedback, collaborating with partners to attend and host community events, maintaining participant ambassador boards, providing curated education and connection through social media, and developing culturally responsive programming. These efforts support four active HAARC Center studies: Communication Bridge 3 Clinical Trial, HAARC Precision Study, ADNI4, and the SuperAging Research Initiative. Our research registry also serves as a platform for engaging individuals interested in research and a tool for fostering strong community connections. Community engagement and recruitment are tracked in REDCap for effective data management and accountability.

**Result:**

The HAARC Center participated in 126 community events and meetings, reaching over 1,500 community members across Chicagoland. Through CER efforts, connections were made with 70 potential participants aged 50 to 92, 80% of whom identify as Black. Older adults comprised 75% of program participants, and 80% of programming focused on underrepresented groups. We established strong partnerships with nine community organizations, 14 institutions and large nonprofits, and seven medical organizations. Our programming includes dementia and healthy aging presentations, Dementia Friendly Communities initiatives, “Ask the Doctor” tables, the ALZ Brain Trust board membership, and memory kit distribution in public libraries. The HAARC Center is the only ADNI Diversity Hub site in Chicago, highlighting leadership in CER and ADRD research.

**Conclusion:**

By fostering partnerships with local organizations, advocacy groups, caregivers, and community members, we ensure that our research remains rooted in lived experiences. Through CER, the HAARC Center strives to improve representation in research, driving equitable advancements in aging and dementia care while reinforcing our commitment to inclusivity and advancing the science of recruitment.

